# Understanding Barriers and Facilitators for Ethnic Minority Groups to Audio Recording Recruitment Discussions in Clinical Trials: A Participatory Approach to Improving Informed Consent and Participation

**DOI:** 10.1111/hex.70210

**Published:** 2025-03-17

**Authors:** Saba Faisal, Giles Birchley, Julia Wade, Athene Lane, Frida Malik, Tom Yardley, Shoba Dawson

**Affiliations:** ^1^ University of Bristol Bristol UK; ^2^ PPIE Contributor Bristol UK; ^3^ University of Sheffield Sheffield UK

**Keywords:** clinical trials, community engagement, ethnic minorities, informed consent, patient and public involvement, recruitment

## Abstract

**Introduction:**

Fully informed consent is essential for ethical trial conduct, yet gaps in participant comprehension and recall can occur, particularly among underserved groups, for example, ethnic minorities. This Patient and Public Involvement and Engagement (PPIE) project explored the engagement of ethnic minority communities in trial recruitment discussions, particularly their views about audio recording discussions with healthcare professionals.

**Methods:**

This PPIE project engaged ethnic minority communities in Bristol, collaborating with community partners to facilitate access to then foster dialogue among Somali, South Asian and Chinese groups. Separate workshops for men and women from these ethnic groups were held to introduce community members to clinical trial processes. Discussions, both audio recorded and not, simulated real recruitment scenarios. To ensure cultural relevance and accessibility, discussions were partly facilitated by our PPIE community partners in native languages.

**Results:**

The insights gained during workshops were organised into key themes. Gaps in understanding regarding clinical trial participation were highlighted. A key finding was that trust played an important role and was facilitated by engaging community leaders and ensuring cultural and linguistic sensitivity during discussions. To address gaps in knowledge about trials and streamline the educational process, we developed storyboards and multilingual video resources. These explained the importance of clinical trials generally and the importance of recruiting diverse patient populations in particular. The materials were co‐created with community partners and refined through iterative feedback to ensure accuracy and cultural appropriateness. The challenge of language barriers necessitated skilled interpreters, especially when discussions were audio recorded, to optimise understanding among people from diverse ethnic backgrounds. The video, available in English, Urdu, Mandarin, Cantonese and Bangla, facilitates understanding of trial purposes and processes, with the aim of widening trial participation in these groups.

**Conclusion:**

Our PPIE activities highlighted gaps in understanding, the critical role of trust and the challenge of language barriers. The co‐created resources have been made available for those wanting to address and overcome some of these issues. The initial feedback from the clinical trials community on the video resources has been promising, underscoring their potential to impact future recruitment efforts and PPIE activities.

**Patient or Public Contribution:**

To foster a co‐creation process, this project included the active involvement of our PPIE collaborators and co‐applicants ‘Khaas’ for funding. They also helped us reach contributors from the South Asian community (mainly of Pakistani and Bangladeshi origin) and arrange workshops. Our two PPIE contributors from Somali Resource Centre and Barton Hill Activity Club helped us reach the Somali community at the Wellspring Settlement. Similarly, the Chinese Community Wellbeing Society helped us reach people from the Chinese community. These PPIE partners also helped us run the workshop by providing live translation of discussion. They also helped translate video scripts and do voiceovers in videos. Also, PPIE contributors Tom Yardley and Amanda Roberts helped with the script development.

## 1 Introduction

Informed consent (IC) is a process that ensures participants' autonomy when they enrol in a clinical trial. The International Conference on Harmonisation (ICH) guidelines for Good Clinical Practice (Section 4.8.10) necessitate that for IC in clinical trials, participants are given complete disclosure of the study specifics and their involvement, they fully comprehend the provided information and are allowed to voluntarily choose to participate [1, 2]. Typically, discussion about potentially taking part in a clinical trial occurs between the participant and the researcher/clinician referred to here as a recruitment discussion. This discussion occurs before signing the IC document and may take place before or after the opportunity to read a Participant Information Sheet (PIS) detailing study information, potential risks and benefits. It allows the potential participant to ask questions and the researcher/clinician to check on participant understanding. However, numerous studies indicate a deficiency in participant comprehension and recall of crucial trial information [3, 4, 5, 6, 7].

A range of obstacles can prevent participants from truly understanding the information they receive during the IC process. An issue raised by Pietrzykowski and Smilowska [8] is that participants might believe that they have understood the provided details, when in reality their grasp is inadequate. Additionally, healthcare professionals may assume that the information they are conveying is easily understandable and adequate when, in some circumstances, this is not the case [8]. However, the ethical principle of patient autonomy in medical research relies on the premise that the IC procedure imparts full awareness to patients/participants about what they are agreeing to [9]. If this assumption cannot be shown to be valid, then there are serious ethical weaknesses in the present system of obtaining consent for clinical trials.

Qualitative research techniques, like observations, interviews and focus groups, have been used to give in‐depth insight into recruitment issues in trials [10]. Although interviews and focus groups are useful for capturing subjective viewpoints and experiences, there can be inconsistencies between actual events and people reported accounts [11]. Based on Jagosh's simplified explanation, these perspectives are only one layer of social reality in the ‘empirical domain’, existing alongside unobserved events and underlying causal mechanisms that are not always immediately apparent [12]. In some cases, qualitative approaches that directly record events and interactions (such as audio recordings, observations and documentary analysis of trial processes) may be more suitable for investigating trial processes [10]. Donovan et al. [13], in the ProtecT randomised trial, analysed audio recordings of recruitment discussions, demonstrating how the language used to present study information impacted participant randomisation. This innovative approach underpinned the development of the QuinteT Recruitment Intervention (QRI), which addresses recruitment difficulties in trials through real‐time analysis and collaborative action plans and has been widely used across trials in the United Kingdom [14].

Since the onset of the COVID‐19 pandemic in the United Kingdom, significant health disparities among ethnic minority communities have become more evident [15]. These groups experienced higher rates of diagnosis [16], severe disease [17] and mortality [18], attributed to factors such as social deprivation, pre‐existing health conditions, large or multigenerational households and limited access to healthcare services [19, 20, 21]. On the other hand, low participation rates in clinical trials by ethnic minority groups have historically limited the scientific applicability and risk detection capabilities of trial findings [22, 23]. The UK trial diversity statistics show that although ethnic minority groups are more likely to be invited to participate in trials (18% vs. 14% for White participants), their participation rate is lower (36% vs. 46% of those invited) [24]. Despite efforts by UK health and social policies to prioritise equality, diversity and inclusivity (EDI) since the 2000s [25], inclusivity remains a challenge in trials research [26], and UK ethnic minorities remain under‐represented in clinical research generally [27, 28]. The continued under‐representation of ethnic minorities in research will further amplify health disparities [29].

Ethnic minorities are reluctant to participate in clinical trials [30, 31], with barriers including mistrust of physicians and research [32, 33], religious beliefs [34, 35], knowledge gaps about trials [36], fear of harm [31] and logistical obstacles such as time and cost [35, 37], which are exacerbated by intersectionality [38]. The persistent lag in the recruitment of ethnic minority groups despite previous reporting of barriers and solutions [39] indicates the complex and enduring nature of this problem. This exclusion operates along ethnic and class lines, ensuring that those who would benefit most from participation in research within ethnic groups are the least likely to participate in it.

Although barriers related to clinical trial recruitment among ethnic minorities are well documented, there is a lack of understanding of barriers relating specifically to audio recording of clinical trial recruitment discussions as used in the QRI. Lack of trust in research has been reported as one of the main concerns among ethnic minority groups, which can translate into declining permission to audio record trial recruitment discussions [40]. Several qualitative methods exist to improve the IC process in trials and include audio recording of recruitment discussions [41, 42]. Therefore, we aimed to understand the facilitators and barriers to consenting to audio recording recruitment discussions within the clinical trial context, which in turn can influence consent to participation in trials.

One strategy to increase the involvement of under‐represented populations in health studies centres on the engagement of patients, the public and local communities throughout the research process [43]. The recent UK National Institute for Health and Care Research (NIHR) INCLUDE guidelines for enhancing representation further recommends building robust community partnerships, collaborative development and empowering groups to shape research priorities and interventions meaningful to them [44]. To understand research needs relevant to ethnic minority groups in the United Kingdom, we employed a Patient and Public Involvement and Engagement (PPIE) approach, thus facilitating their direct input and participation.

## 2 Materials and Methods

### 2.1 Population

This PPIE project was incorporated into S. F.'s PhD project, investigating ways of optimising information provision during trial recruitment discussions. According to the 2021 UK Census data for Bristol, it is becoming an increasingly diverse city, with 28.4% of its population comprising of 18 ethnic minority groups, with the largest proportion being Somali, South Asian and Chinese ethnicities [45]. Therefore, we conducted workshops among these ethnic minority communities.

### 2.2 PPIE Collaborators and Contributors

To foster a co‐creation process, this project included from its inception the active involvement of our PPIE collaborators and co‐applicants for funding, ‘Khaas’. Khaas is a community‐based third sector organisation that has worked across the Southwest and the Bristol area, delivering health, educational and social services to improve the lives of ethnic minorities and disabled children, their carers and families for over 35 years. They also helped us reach contributors from the South Asian community (mainly of Pakistani and Bangladeshi origin) and arrange workshops.

Our two PPIE contributors, Zahra Kosar and Samira Musse, helped us reach the Somali community via The Friday Coffee Morning, which is a weekly, community‐run event at the Wellspring Settlement. The meeting gathers about 10–15 local women, predominantly of Somali background, to socialise. Similarly, the Chinese Community Wellbeing Society helped us reach people from the Chinese community. The description of workshops and contributors that attended the discussion can be seen in Table 1.

**Table 1:** Workshop description.

Community	Contributors	Venue	Date of workshop
South Asian	15 women (11 Pakistani, 3 Bengali and 1 Indian) 8 men (all Pakistani)	St Werburghs Community Centre, Bristol	19 May 2023 (women) 12 June 2023 (men)
Somali	16 men and 15 women	University of Bristol's Barton Hill microcampus within the Wellspring Settlement, Bristol	18 May 2023 (men) 26 May 2023 (women)
Chinese	22 men and women	Vassall Centre, Bristol	27 June 2023

Connecting with community leaders was an essential first step. The goal was to establish links and develop relationships to facilitate introductions and advocacy. Building rapport entailed direct in‐person meetings, supplemented by phone calls and emails, to discuss preferred approaches to involvement activities. The discussion format and interactions were customised based on the needs and inclinations of each distinct community group, with open‐ended, flexible conversations adapted to best suit different preferences.

### 2.3 Engagement and Workshops

**Figure 1:**
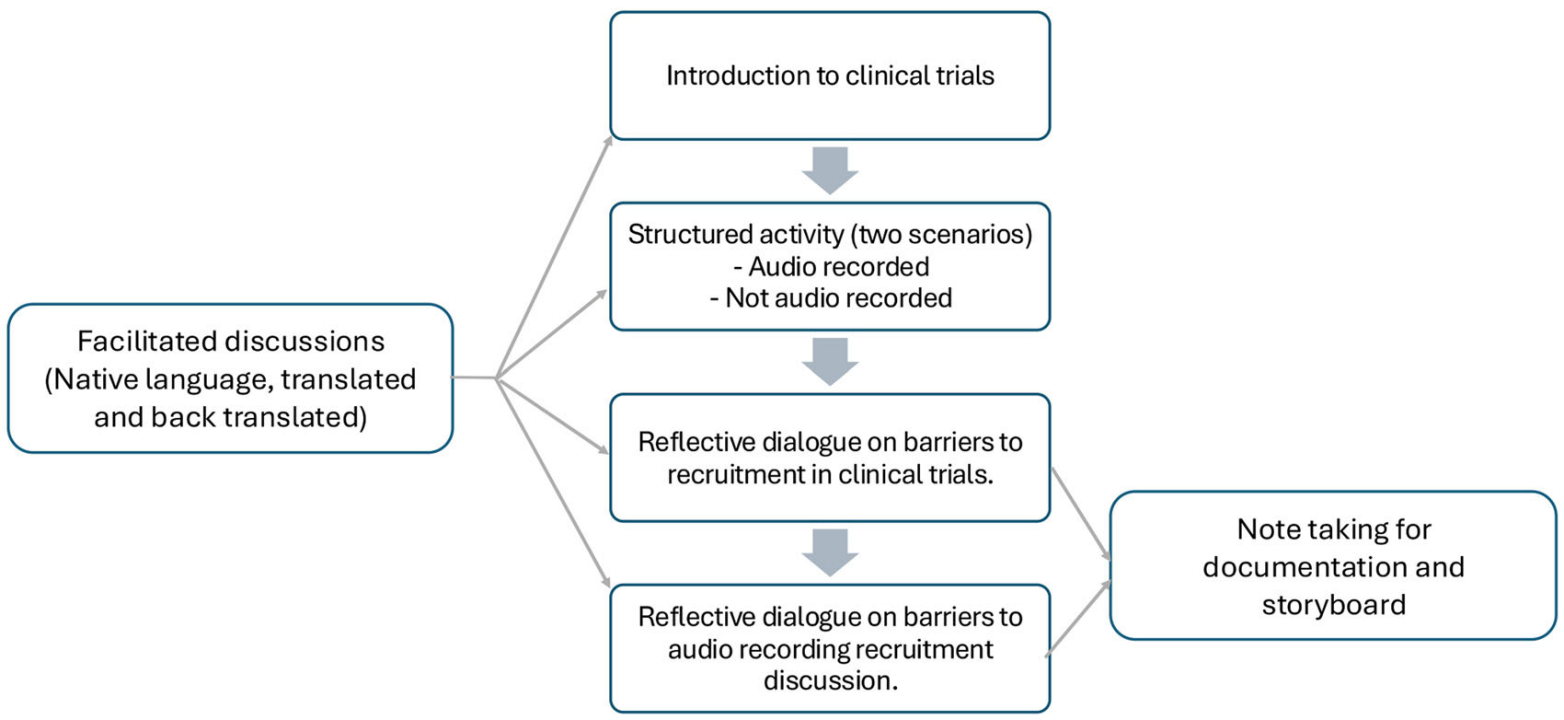
Flow chart explaining workshop structure.

Initially, an online workshop was conducted with individuals who were PPIE contributors in clinical trials in March 2023. They were recruited via trial teams through newsletters and NIHR Bepartofresearch. The discussion in this online workshop provided us with a deeper understanding of the related issues and guided in drafting questions for further workshops. The second set of workshops were with individuals from ethnically diverse communities under‐represented in trials. For cultural reasons, separate workshops were held for men and women in Somali and South Asian communities. The structure of workshops is also summarised in Figure 1.

In the workshops, PPIE contributors were first presented with information on the purpose and structure of clinical trials. Subsequently, contributors actively engaged in a structured activity designed to stimulate discussions on a given topic. Notably, this activity involved two distinct conditions: one where contributors were audio recorded and another where no such recording took place. The audio recordings stemming from the activity were deleted at the end of each workshop, as the primary objective of this exercise was to immerse contributors in an experience.

Following this, contributors were encouraged to engage in a reflective dialogue regarding the obstacles and reservations they might have regarding the acceptance of audio recordings. Discussions were facilitated partially in the communities' native languages to promote open idea‐sharing and organically foster rapport. Community partners translated exchanges to retain clarity.

All discussions pertaining to participation in clinical trials and matters of trust concerning audio recording were documented through note taking. Contributors were notified that written notes would capture these discussions for transparency. The rationale for nonintrusive logging was to not interrupt the natural environment or influence the conversation. By not transcribing conversations directly and making the purpose of notation clear, the aim was to strike a balance between accurately recording the rich dialogue and building an accepting atmosphere founded on trust.

Contributors, as well as our community partners, were remunerated for their contributions and time based on the NIHR Centre for Engagement and Dissemination guidance [46].

### 2.4 Storyboard and Video Outputs

**Figure 2:**
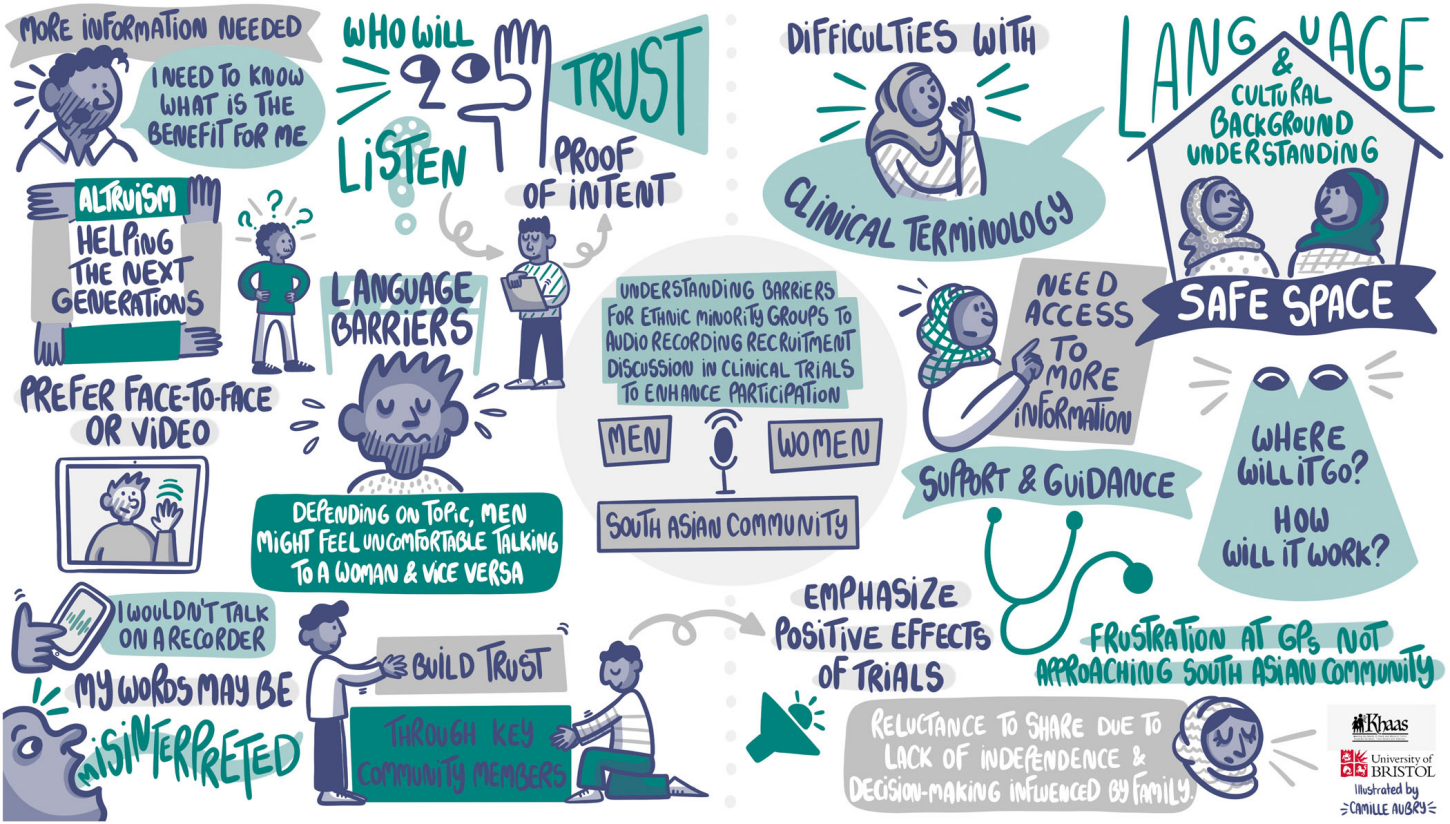
Storyboard for discussion with the South Asian community.

**Figure 3:**
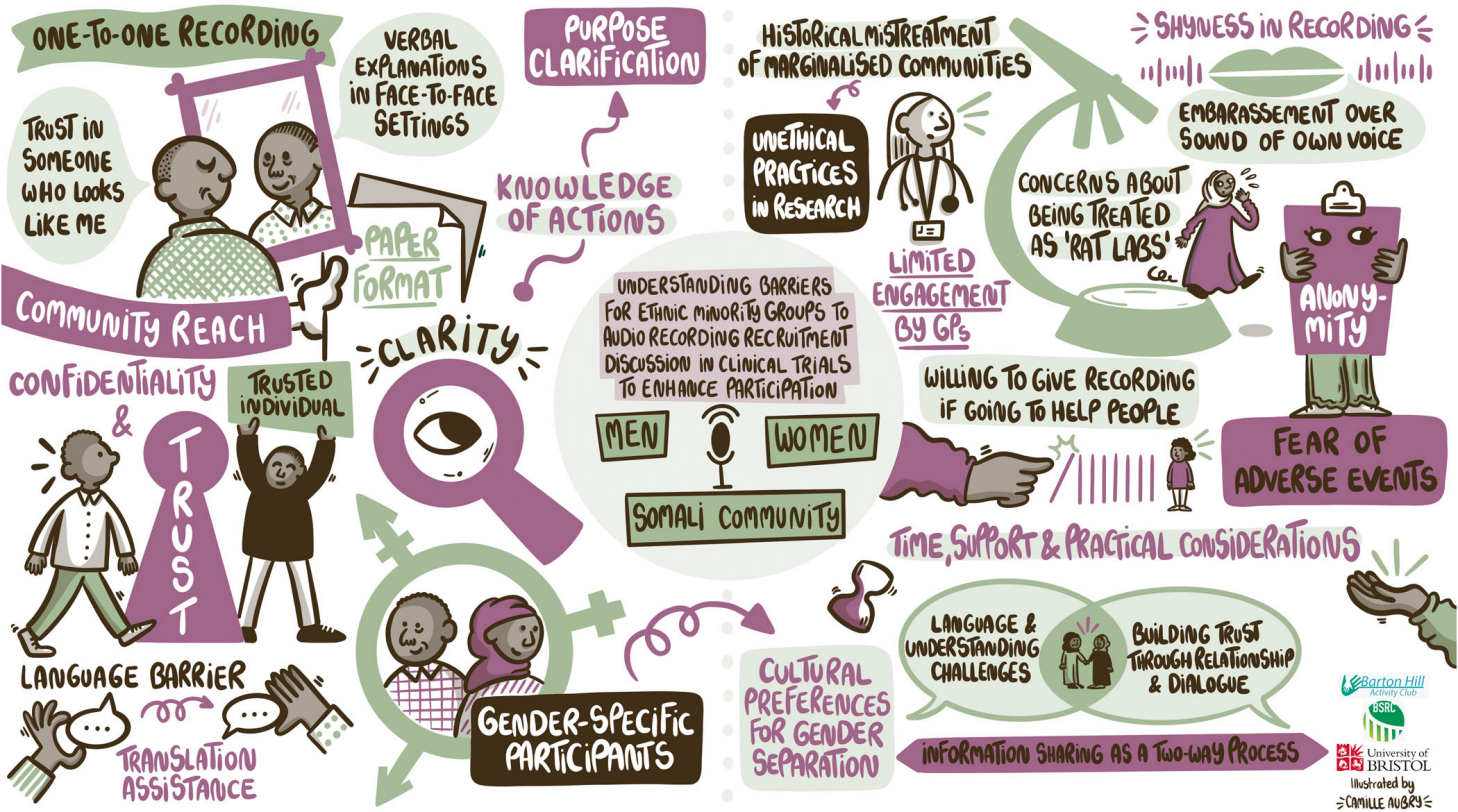
Storyboard for discussion with the Somali community.

**Figure 4:**
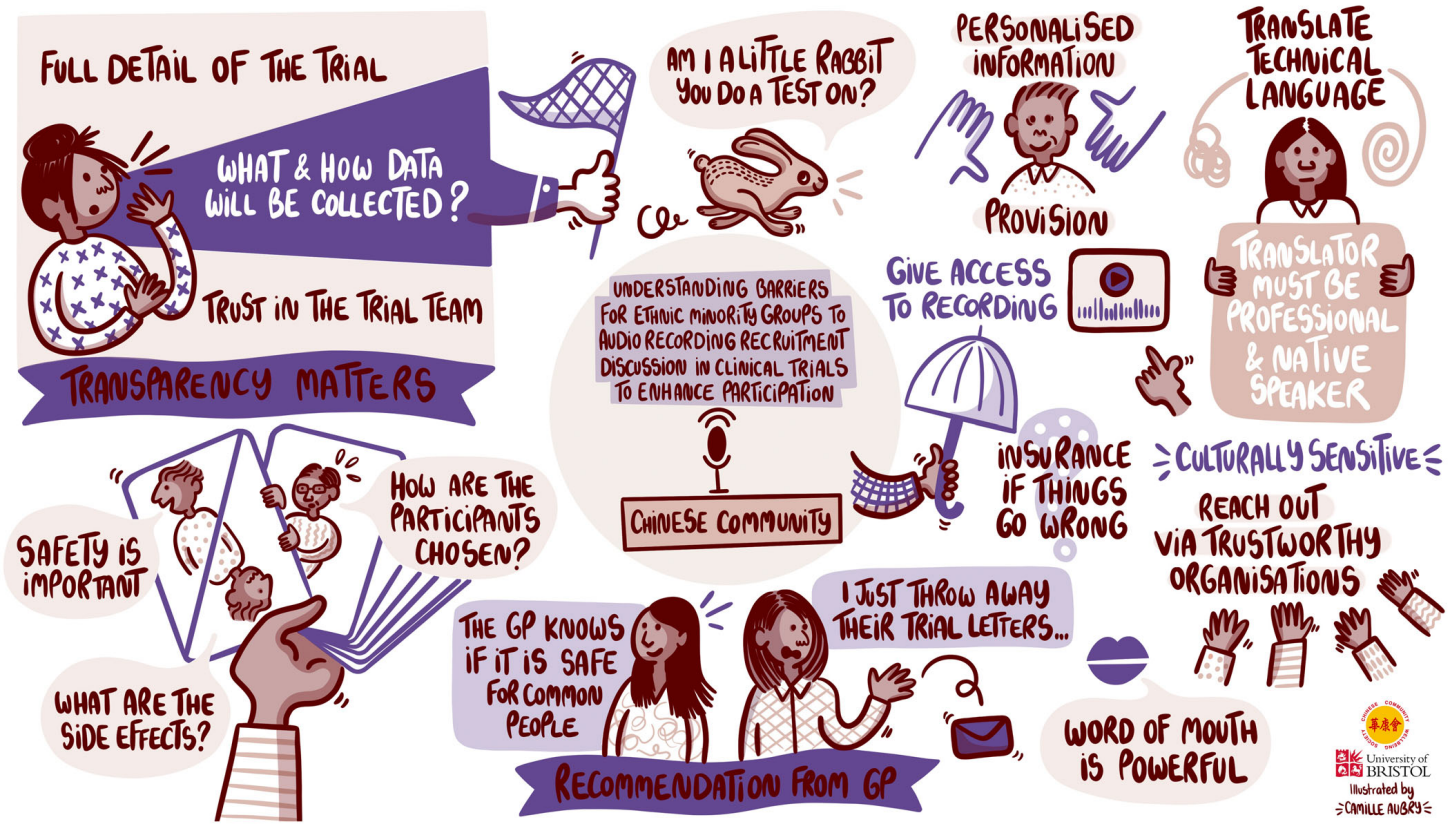
Storyboard for discussion with the Chinese community.

We created three storyboards of discussions from the workshops (one for each community) by working with an illustrator (Figures 2, 3, 4). The illustrator was provided with the notes, key themes and photos from the community group discussions, which she turned into a draft of a visual illustration of key themes. We worked with our PPI co‐applicant and other PPI contributors to ensure that no information was missing or misrepresented, and content was presented in a culturally sensitive and acceptable manner. As a result, the final illustration used plain language and appropriate terminology. It served as an output for both the communities and external audiences, conveying insights while maintaining an accessible community lens.

We also aimed to create a video resource that can be used during other PPIE discussions or trial recruitment to raise awareness about clinical trials amongst potential contributors. This arose from discussions with both PPI co‐applicant and through the workshops. Building upon the dialogues held during the workshop sessions and in collaboration with our PPI contributors, we co‐produced a short video in five languages: English, Urdu, Mandarin, Cantonese and Bangla. The video script went through various iterations of screening and editing, including input from members of the Trials Methodology Research Partnership (TMRP) inclusivity working group and our PPIE collaborators to ensure the language was simple and free of jargon. To ensure translation accuracy, each video script was reviewed by two native speakers in which one was someone with a medical background and another was a lay member to ensure medical terminologies were accurately translated but at the same time translations were not too complex to understand. This script and later the video was then sent to community partners to ensure cultural appropriateness, clarity and relevance. This video addressed fundamental questions related to clinical trials and the rationale behind the practice of audio recording recruitment discussions.

### 2.5 Analysis

No formal qualitative analysis such as thematic or framework analysis was employed in this PPIE project. The written notes were organised into an Excel sheet for a structured examination. Each row represented the notes from one community, whereas columns were created to group together related points under corresponding themes. The content of each column was then summarised and critically evaluated to identify key insights and perspectives.

### 2.6 PPIE Reporting

This manuscript reports the PPIE work in alignment with the GRIPP2 methodology that provides standardised reporting items to improve the quality, transparency and consistency of PPIE work within health studies [47]. A completed GRIPP2 short‐form checklist summarising our PPIE approach can be found in Supporting Information Appendix 1.

## 3 Results

The following six themes were identified in the discussions about consent to take part in a trial and audio recording of recruitment discussion.

### 3.1 Building Trust in Research

The most common issue discussed during the workshops was how trust can be built with these communities during recruitment. Language emerged as a significant factor for trust building. Contributors believed that accessible information in their native language would be received well. They cautioned against relying solely on automated tools, so professional translators fluent in medical terminology were crucial to communicate concerns effectively.

Translator is needed so I can explain my concern. (Workshop 1, Somali men)

Using plain language and having community representatives were deemed essential for comprehension and confidence in recruitment discussions. Having people from the same cultural background on the trial team would also encourage participation.

More people will join the trials once some people understand – more approachable if we already know people on the trial. (Workshop 4, South Asian men)

Moreover, Somali contributors were more hesitant when the trial involved children, especially as part of random allocation. This caused contributors to ask ‘why [am I] getting different treatment?’ which in their opinion increased their deep‐rooted mistrust of research related to historical mistreatment. They referred to the Tuskegee experiment to explain their concern, even though they were not directly impacted by it, they remembered how their communities have been treated in the past.

Chinese community partners asked what insurance and liability are given if harm occurs, explaining insurance would increase their trust. They expected their recruiter to be honest with them about potential treatment risks, which would show their respect and care. For them, a convincing answer to ‘Why should I join?’ was grounded in trust, not monetary or any other personal gain. They mentioned that with care and understanding from researchers, sceptical questions can become an opportunity to build trust through dialogue.

### 3.2 Views on Audio Recording Recruitment Discussions

The discussion about audio recording of recruitment discussion varied among the three communities. The South Asian women admitted to censoring or withholding their concerns and opinions when recorded (during mock discussion). Similarly, South Asian men were not willing to openly discuss their personal health matters when recorded. They discussed their fear of misrepresentation in case someone hears part of the discussion without knowing the full context. Therefore, they mentioned that they would be very cautious in how they phrased their statements, which might interrupt a candid dialogue.

My words may be misinterpreted – ‘only you know what you mean’ i.e. it is possible to misspeak – if recorded you feel careful of what you say. (Workshop 4, South Asian men)

In contrast, Somali men were comfortable doing recorded discussions mainly in a one‐to‐one setting with doctors and nurses. Some community partners from Somali women mentioned: ‘Sound of my own voice is embarrassing’ (Workshop 3). Therefore, they showed interest in receiving written notes or verbatim of the discussion for their record rather than the actual recording.

Unlike the other two community groups, most Chinese community members were quite open to audio recording, with most being comfortable if they were granted access to the recording afterwards.

No major concerns about audio recording – though it is a good idea if patient is then given access to audio recording. (Workshop 5, Chinese community member)

They reasoned that the provision of audio recording would demonstrate transparency and be suitable for record‐keeping if they needed to recall what was discussed and agreed upon. Some mentioned that agreeing to audio recording is conditional to the value of the trial and the integrity of the trial team.

It was a common argument among all three groups that they were wary of how their recordings would be used and by whom beyond the recruiters. They needed clarity about handling of and access to recorded data.

### 3.3 Ethical Safeguards

In the South Asian community discussion, it was highlighted that they feared the presence of an interpreter for translation as they might know them from the community and therefore there could be a breach of confidentiality of their recruitment discussion. Especially the older generation was sceptical about other people from the wider community knowing of their involvement in research. This was especially the case for health conditions which could be sensitive in nature. Both Somali and Chinese community members largely felt apprehensive about the selection method and worried they would be treated like ‘lab rats’ or ‘test subjects’. Therefore, understanding the trial focus, risks and benefits of treatment was highly important for them.

All community contributors stressed the importance of full transparency in trial procedures. They needed to know in advance what data would be collected, why and how their privacy would be safeguarded.

### 3.4 Missed Opportunities

An interesting discussion emerged among the South Asian and Somali women. They discussed their frustration over the lack of outreach from the general practitioners (GPs) in encouraging or informing them about clinical trials. They argued that if GPs did not inquire about their interests in taking part in studies, this would limit their opportunity to get involved. Some defended the GPs by arguing that with increased demand on healthcare practitioners, there is limited time in the ‘10 min slot’ to address ‘research’. They talked about their lack of awareness about trials relevant to them and their difficulty in finding information.

Some South Asian women mentioned that they felt minimal effort has been made to include them in research, even when there is no language barrier, and they can communicate well in English.

Language barrier for some people but we don't get approached at all. (Workshop 2, South Asian women)

Overall, these women groups also highlighted some barriers to taking part in trials that were not mentioned in male groups, such as time constraints, the need to prioritise family matters, the voluntary nature of the study, childcare support and transport.

### 3.5 Altruism as a Motivating Factor

Altruism was a common motivation found in all discussions. For the South Asian community, despite their religious concerns and uncertainties about experimental drug compositions, there was a commitment to support medical research for the greater good and the well‐being of future generations. Similarly, Chinese and Somali communities also emphasised that they were interested in research that could help others and is meaningful. The common sentiment that echoed was:

If it's going to help someone then why not.

### 3.6 Cultural Factors

Some unique cultural factors among each community highlighted their concerns in relation to participation in clinical trials and/or consenting to audio recording.

South Asian women voiced different concerns compared to men, which included a lack of independence in making decisions regarding their health and the significant influence of family members in decision‐making. Some mentioned difficulties they would face in convincing men in their families if they wanted to participate, pointing out men's different priorities and mindsets compared to them. They were also concerned about certain medications and procedures which might not comply with their religious dietary restriction. Another added concern was the complexity of explaining traditional cultural practices to researchers.

Meanwhile, South Asian men highlighted issues related to gender. They acknowledged their discomfort in discussing topics of men's health like prostate cancer screening with female physicians or researchers. We highlighted the issues raised by women in their discussion related to being dependant on families for their decisions. These men criticised their male peers and discouraged the influence of men on women's health decisions, stressing that women should have been independent in making their choice. Somali men and women advised aligning same‐sex recruiter and translator when recruiting for sensitive subjects, whereas, for general health issues, the gender of the recruiter was not important. They accentuated that gender‐based segregation aligns with their religious and cultural preferences.

Generic topic who discusses does not matter. If it is a sensitive topic, match men with men and women with women. Same for translators as well. (Workshop 1, Somali men)

Due to stigma around certain issues like organ donation, they emphasised the need for guidance from their religious leaders to help them align their religious beliefs and values when participating in such trials. Candid discussion with the recruiters would also help in deciding how such trials fits with their religious and cultural beliefs.

In contrast, Chinese community partners discussed their culture rooted in Chinese family‐based care, contrasting with the individual focus in the UK healthcare system. For them, discussing with their family was a priority before making any healthcare decision. Hence, involving family members in the recruitment discussion could be another facilitating approach.

Family members communicate to direct care – it is difficult to get UK culture to accept family basis of care. (Workshop 5, Chinese community member)

Furthermore, the positive experiences of peers can highly influence decision‐making. As in the South Asian community, in the Chinese community, reluctance from male family members could cause obstacles. However, they suggested that recruiters should recognise and accommodate Chinese communal decision‐making.

### 3.7 Value of Approach

From the discussions, it was evident that the contributors valued how they were approached, and this was related to a positive or negative response to trial recruitment.

For South Asian men, if the trial team approached them through community‐trusted institutions or religious institutions (mainly mosques and Imams), the chances of their participation would increase. Because they were Muslim, South Asian contributors wanted reassurance about trial drug contents (such as non‐Halal ingredients) and safety, which they thought the religious institutes could help them verify. They believed it would not matter whether the recruitment process was recorded if they were approached via a trusted organisation. For South Asian contributors, advertisements on Asian TV channels and explanatory videos could also boost understanding, as many find visuals easier than text, whereas, some preferred face‐to‐face interactions to build trust by assessing recruiters' sincerity.

For the Somali community, meeting people already participating in the trial would help in making decisions by having first‐hand knowledge of good and bad experiences. They registered their resistance by saying, ‘I am not a lab rat’, and condemned being treated solely as a research subject. They emphasised being approached in a welcoming, respectful manner and establishing a sense of partnership. They wanted their recruiter or consent taker to know the subject matter so that they could get sufficient information and discuss the risks and benefits of the study.

For the Chinese community, information about the trial was most trusted if their GPs recommended it or if they were familiar with the trial team. The GP's assurance would ensure the trial's legitimacy and boost their confidence in participation. However, they disapproved of generic letters from the GPs, broad advertisements and mass mailing.

Usually I just throw away a letter from the GP for a trial. (Workshop 5, Chinese community member)

For them, personalised discussion was the preferred method of approach. They believed it would allow them to ask questions, understand the trial and build rapport with the team.

## 4 Discussion

For many of us authors, conducting PPIE was a new experience. However, with guidance from the experienced member (S. D.), we ensured that trusting relationships were built with the PPIE partners, and a safe space was provided to the contributors for an open discussion [48].

Across all these discussions, the major emerging theme was building trust via approaching community leaders or influencers. Lack of trust and limited access to trials among underserved groups in research were the barriers also identified in a rapid review by Bodicoat et al. [49]. There have been several studies that have successfully used the strategy of engaging with communities to build trust by providing educational sessions for them [50, 51, 52, 53, 54, 55, 56]. A PPIE study involving Bristol researchers and community organisations working with South Asian, African Caribbean and Somali groups also emphasised the importance of building trust [57]. Their co‐produced CHecklist for Inclusive COmmunity involvement in health research (CHICO) highlights engaging community leaders and understanding their groups as essential first steps in PPIE.

Language and cultural sensitivity were other common emerging themes. People were keen to participate in clinical trials; however, they needed to be assured that their cultural preferences would be accommodated. Another PPIE study also emphasised the importance of cultural sensitivity, recommending that healthcare professionals and staff, receive cultural sensitivity training to account for cultural context in patient interactions [58]. This key theme is also a recommendation in *Trial Forge Guidance 3* [59], which highlights importance of language, translation and cultural appropriateness when developing trial materials to promote inclusivity.

For those facing a language barrier, the presence of a professional interpreter during recruitment discussions was highly encouraged, especially when the discussions were to be audio recorded. Since audio recordings serve as evidence of the discussion, contributors wanted to be certain they completely understand every detail, without confusion due to language. A skilled interpreter could ensure comprehension of the given information. However, it should be noted that due to resource constraints, arranging interpreters is not always possible, and the language barrier continues to be the biggest barrier for many professional researchers in recruiting people from ethnic minorities [60].

Furthermore, during our workshops, we needed to spend a substantial amount of time explaining a clinical trial to the contributors, as there were misconceptions and a lack of understanding in terms of what a trial involved. This task was crucial, as we needed it to be able to initiate the discussion. We had to clarify to the contributors what a clinical trial was and guide them through it. However, if we had a short video, presented in their language, which takes them through the process, it would have made the task much more straightforward. A preference for educational resources in the form of video was highlighted in the group discussions. Therefore, we created a video resource of approx. 5 min that can be used during other PPIE discussions or trial recruitment to answer basic questions related to clinical trials such as what are clinical trials; why participate; diversity and inclusion; recruitment process; participants' rights and IC, and reasons for audio recording recruitment discussion. It is free to use and available online at (https://t.co/9nGR5xd3Vr). The message in the video has been delivered by native speakers to resonate with the community.

These videos aim to serve as an educational tool to disseminate critical insights to a broader audience in future clinical trials, especially when working with diverse communities to facilitate research participation. When used in other PPIE events, this video can be cost‐effective, as it has the potential to save time for individuals involved. Furthermore, it can help potential study participants make informed decisions when participating. These outputs can be shared via social media, the QuinteT website, TMRP groups, and future trial dissemination and PPI events with various community organisations.

There were some limitations of the project. It was difficult to recruit South Asian men. The reason could be working hours (as meetings were held during the day), lack of interest or weakness of our approach to reaching them. From South Asian community, all PPI male contributors were from British Pakistani ethnicity. Fewer Pakistani men attended because, while Khaas is predominant in the Pakistani community, it is run by women who, for cultural reasons, have less reach to men than women. For future engagement, it is important to consider approaching men through mosques or other cultural or religious places and hosting the event in the evening or on a non‐working day to help in their recruitment. To better engage South Asian men in future PPIE work and facilitate wider research participation, it is crucial to educate existing male community leaders about PPIE, as current contributors are predominantly women and mosques only target men who attend them. We recommend that efforts be made to encourage inclusion of more male members of South Asian ethnicity in research discussions as their perception also influence the recruitment of women and kids in their family.

Other limitations include the inability to contact people who participated in the discussion again. As we wanted contributors to feel safe, no personal data were collected, which prevented us from reaching the same group for further discussion. Although there are eighteen minority groups in Bristol, due to the limited timeframe to complete the project, only the three largest groups were approached.

Our videos have been shared with the clinical trials community for project evaluation, and their feedback will be collected. Based on the feedback, we will focus on the impact and feasibility of the video in the recruitment process.

## 5 Conclusion

In conclusion, this project demonstrated using PPIE effectively enabled open discussions with ethnic minority groups around trust related to audio recording recruitment discussions in clinical trials. Although centring on trust, the emerging key points included steps to help build trust in research. Mainly, full transparency about the trial process, respecting cultural preferences, approaching trusted institutions and partnering with community organisations can improve participation from ethnic minorities in the trials in the United Kingdom. Our findings demonstrate PPIE's capacity to elicit diverse perspectives, identify critical factors influencing consent and meaningfully include under‐represented voices to improve recruitment practices. This collaborative engagement approach gave three ethnic minorities a platform to voice their needs, concerns and preferences regarding consent and audio recording. By focusing on communities' insights, we gained valuable direction in building trust and enhancing consent processes in a culturally conscious manner. This model of participatory engagement can guide future efforts to make clinical trial recruitment more equitable and inclusive.

## Author Contributions

**Saba Faisal:** conceptualization, methodology, data curation, investigation, validation, formal analysis, funding acquisition, visualization, writing – original draft, writing – review and editing. **Giles Birchley:** writing – review and editing, supervision, investigation. **Julia Wade:** writing – review and editing, supervision, investigation. **Athene Lane:** writing – review and editing, supervision, investigation. **Frida Malik:** funding acquisition, writing – review and editing, investigation. **Tom Yardley:** writing – review and editing, investigation. **Shoba Dawson:** writing – review and editing, funding acquisition, supervision, conceptualization, methodology, investigation.

## Ethics Statement

The authors have nothing to report.

## Consent

The authors have nothing to report.

## Conflicts of Interest

The authors declare no conflicts of interest.

## Supporting information

Supporting information.

## Data Availability

The authors have nothing to report.

## References

[1] “IRB Informed Consent,” Research & Innovation, accessed December 31, 2023, https://researchservices.cornell.edu/resources/irb-informed-consent.

[2] ICH Steering Committee “ICH Harmonised Tripartite Guideline: Guideline for Good Clinical Practice E6 (R1)”. (ICH Steering Committee, 1996).

[3] J. Commission , “Informed Consent: More Than Getting a Signature,” Quick Safety 21 (2016): 1–3.

[4] J. Matiasek and M. K. Wynia , “Reconceptualizing the Informed Consent Process at Eight Innovative Hospitals,” Joint Commission Journal on Quality and Patient Safety 34, no. 3 (2008): 127–137. 18419042 10.1016/s1553-7250(08)34015-x

[5] A. Sherlock and S. Brownie , “Patients' Recollection and Understanding of Informed Consent: A Literature Review,” ANZ Journal of Surgery 84, no. 4 (2014): 207–210. 24812707 10.1111/ans.12555

[6] P. Kinnersley , K. Phillips , K. Savage , et al., “Interventions to Promote Informed Consent for Patients Undergoing Surgical and Other Invasive Healthcare Procedures,” Cochrane Database of Systematic Reviews 2013, no. 7 (2013): CD009445. 23832767 10.1002/14651858.CD009445.pub2 PMC11663509

[7] M. Brezis , S. Israel , A. Weinstein‐Birenshtock , P. Pogoda , A. Sharon , and R. Tauber , “Quality of Informed Consent for Invasive Procedures,” International Journal for Quality in Health Care 20, no. 5 (2007): 352–357. 10.1093/intqhc/mzn025 18625699

[8] T. Pietrzykowski and K. Smilowska , “The Reality of Informed Consent: Empirical Studies on Patient Comprehension—Systematic Review,” Trials 22 (2021): 57. 33446265 10.1186/s13063-020-04969-w PMC7807905

[9] A. P. Minei and S. O. Kaipu , “Respect for Patients' Right to Autonomy,” Journal of Health Science 8 (2020): 100–112.

[10] M. Hennessy , A. Hunter , P. Healy , S. Galvin , and C. Houghton , “Improving Trial Recruitment Processes: How Qualitative Methodologies Can Be Used to Address the Top 10 Research Priorities Identified Within the Priority Study,” Trials 19 (2018): 584. 30359293 10.1186/s13063-018-2964-1 PMC6202834

[11] G. Guest , E. Namey , J. Taylor , N. Eley , and K. McKenna , “Comparing Focus Groups and Individual Interviews: Findings From a Randomized Study,” International Journal of Social Research Methodology 20, no. 6 (2017): 693–708.

[12] J. Jagosh , “Realist Synthesis for Public Health: Building an Ontologically Deep Understanding of How Programs Work, for Whom, and in Which Contexts,” Annual Review of Public Health 40, no. 1 (2019): 361–372. 10.1146/annurev-publhealth-031816-044451 30633712

[13] J. Donovan , P. Little , N. Mills , et al., “Quality Improvement Report Improving Design and Conduct of Randomised Trials by Embedding Them in Qualitative Research: Protect (Prostate Testing for Cancer and Treatment) Study Commentary: Presenting Unbiased Information to Patients Can Be Difficult,” BMJ 325, no. 7367 (2002): 766–770. 12364308 10.1136/bmj.325.7367.766 PMC1124277

[14] J. L. Donovan , L. Rooshenas , M. Jepson , et al., “Optimising Recruitment and Informed Consent in Randomised Controlled Trials: The Development and Implementation of the Quintet Recruitment Intervention (QRI),” Trials 17, no. 1 (2016): 283. 27278130 10.1186/s13063-016-1391-4 PMC4898358

[15] K. Khunti , A. K. Singh , M. Pareek , and W. Hanif , “Is Ethnicity Linked to Incidence or Outcomes of Covid‐19?,” British Medical Journal Publishing Group 369 (2020): m1548. 10.1136/bmj.m1548 32312785

[16] R. H. Mulholland and I. P. Sinha , “Ethnicity and COVID‐19 Infection: Are the Pieces of the Puzzle Falling Into Place?,” BMC Medicine 18 (2020): 206. 32605617 10.1186/s12916-020-01669-9 PMC7326620

[17] “COVID‐19: Review of Disparities in Risks and Outcomes,” Public Health England, accessed 21 May, 2024, https://www.gov.uk/government/publications/covid-19-review-of-disparities-in-risks-and-outcomes.

[18] D. Pan , S. Sze , J. S. Minhas , et al., “The Impact of Ethnicity on Clinical Outcomes in COVID‐19: A Systematic Review,” EClinicalMedicine 23 (2020): 100404. 32632416 10.1016/j.eclinm.2020.100404 PMC7267805

[19] M. S. Razai , H. K. Kankam , A. Majeed , A. Esmail , and D. R. Williams , “Mitigating Ethnic Disparities in Covid‐19 and Beyond,” BMJ 372 (2021): m4921. 33446485 10.1136/bmj.m4921

[20] J. T. Chen and N. Krieger , “Revealing the Unequal Burden of COVID‐19 By Income, Race/Ethnicity, and Household Crowding: US County Versus Zip Code Analyses,” Journal of Public Health Management and Practice 27, no. suppl. 1 (2021): S43–S56. 32956299 10.1097/PHH.0000000000001263

[21] S. V. Katikireddi , S. Lal , E. D. Carrol , et al., “Unequal Impact of the COVID‐19 Crisis on Minority Ethnic Groups: A Framework for Understanding and Addressing Inequalities,” Journal of Epidemiology and Community Health 75, no. 10 (2021): 970–974. 33883198 10.1136/jech-2020-216061 PMC8458062

[22] J. Roberts , S. Waddy , and P. Kaufmann , “Recruitment and Retention Monitoring: Facilitating the Mission of the National Institute of Neurological Disorders and Stroke (NINDS),” Journal of Vascular and Interventional Neurology 5, no. 1.5 (2012): 14–19. 23230460 PMC3517027

[23] H. K. Sanoff , D. J. Sargent , E. M. Green , H. L. McLeod , and R. M. Goldberg , “Racial Differences in Advanced Colorectal Cancer Outcomes and Pharmacogenetics: A Subgroup Analysis of a Large Randomized Clinical Trial,” Journal of Clinical Oncology 27, no. 25 (2009): 4109–4115. 19636001 10.1200/JCO.2009.21.9527 PMC2734422

[24] “Diversity and Clinical Trials in the UK,” IPSOS, accessed January 15, 2025, https://www.ipsos.com/sites/default/files/ct/publication/documents/2024-02/Health%20Equity_Clinical%20Trial%20Research_Feb2024.pdf.

[25] “Equality, Diversity and Inclusion Strategy 2020–2025,” Companies House, accessed May 21, 2024, https://assets.publishing.service.gov.uk/government/uploads/system/uploads/attachment_data/file/1120675/EDI_strategy_Companies_House.pdf.

[26] V. Nanton , R. T. Bryan , A. M. Pope , et al., “Boosting and Broadening Recruitment to UK Cancer Trials: Towards a Blueprint for Action,” BMJ Oncology 2 (2023): e000092. 10.1136/bmjonc-2023-000092 PMC11235001 39886495

[27] S. Treweek , K. Banister , P. Bower , et al., “Developing the INCLUDE Ethnicity Framework—A Tool to Help Trialists Design Trials That Better Reflect the Communities They Serve,” Trials 22, no. 1 (2021): 337. 33971916 10.1186/s13063-021-05276-8 PMC8108025

[28] G. Catney , C. D. Lloyd , M. Ellis , et al., “Ethnic Diversification and Neighbourhood Mixing: A Rapid Response Analysis of the 2021 Census of England and Wales,” Geographical Journal 189, no. 1 (2023): 63–77.

[29] M. S. Chen , P. N. Lara , J. H. T. Dang , D. A. Paterniti , and K. Kelly , “Twenty Years Post‐NIH Revitalization Act: Enhancing Minority Participation in Clinical Trials (EMPaCT): Laying the Groundwork for Improving Minority Clinical Trial Accrual: Renewing the Case for Enhancing Minority Participation in Cancer Clinical Trials,” Cancer 120 (2014): 1091–1096. 24643646 10.1002/cncr.28575 PMC3980490

[30] M. Hussain‐Gambles , K. Atkin , and B. Leese , “Why Ethnic Minority Groups Are Under‐Represented in Clinical Trials: A Review of the Literature,” Health & Social Care in the Community 12, no. 5 (2004): 382–388. 15373816 10.1111/j.1365-2524.2004.00507.x

[31] G. Y. Lai , T. L. Gary , J. Tilburt , et al., “Effectiveness of Strategies to Recruit Underrepresented Populations Into Cancer Clinical Trials,” Clinical Trials 3, no. 2 (2006): 133–141. 16773955 10.1191/1740774506cn143oa

[32] R. D. Branson , K. Davis, Jr. , and K. L. Butler , “African Americans' Participation in Clinical Research: Importance, Barriers, and Solutions,” American Journal of Surgery 193, no. 1 (2007): 32–39. 17188084 10.1016/j.amjsurg.2005.11.007

[33] J. G. Ford , M. W. Howerton , G. Y. Lai , et al., “Barriers to Recruiting Underrepresented Populations to Cancer Clinical Trials: A Systematic Review,” Cancer 112, no. 2 (2008): 228–242. 18008363 10.1002/cncr.23157

[34] S. Daverio‐Zanetti , K. Schultz , M. A. M. del Campo , V. Malcarne , N. Riley , and G. R. Sadler , “Is Religiosity Related to Attitudes Toward Clinical Trials Participation?,” Journal of Cancer Education 30 (2015): 220–224. 24953236 10.1007/s13187-014-0696-9 PMC4276542

[35] D. Rivers , E. M. August , I. Sehovic , B. Lee Green , and G. P. Quinn , “A Systematic Review of the Factors Influencing African Americans' Participation in Cancer Clinical Trials,” Contemporary Clinical Trials 35, no. 2 (2013): 13–32. 23557729 10.1016/j.cct.2013.03.007

[36] A. R. Giuliano , N. Mokuau , C. Hughes , et al., “Participation of Minorities in Cancer Research,” Annals of Epidemiology 10, no. 8 (2000): S22–S34. 11189089 10.1016/s1047-2797(00)00195-2

[37] S. George , N. Duran , and K. Norris , “A Systematic Review of Barriers and Facilitators to Minority Research Participation Among African Americans, Latinos, Asian Americans, and Pacific Islanders,” American Journal of Public Health 104, no. 2 (2014): e16–e31. 10.2105/AJPH.2013.301706 PMC3935672 24328648

[38] U. A. Kelly , “Integrating Intersectionality and Biomedicine in Health Disparities Research,” Advances in Nursing Science 32, no. 2 (2009): E42–E56. 10.1097/ANS.0b013e3181a3b3fc 19461221

[39] J. M. Watson and D. J. Torgerson , “Increasing Recruitment to Randomised Trials: A Review of Randomised Controlled Trials,” BMC Medical Research Methodology 6 (2006): 34. 16854229 10.1186/1471-2288-6-34 PMC1559709

[40] S. Dawson , A. Ruddock , V. Parmar , et al., “Patient and Public Involvement in Doctoral Research: Reflections and Experiences of the Ppi Contributors and Researcher,” Research Involvement and Engagement 6 (2020): 23. 32426162 10.1186/s40900-020-00201-w PMC7216324

[41] J. Wade , E. Humphrys , A. X. Realpe , et al., “Informed Consent in Randomised Controlled Trials: Further Development and Evaluation of the Participatory and Informed Consent (PIC) Measure,” Trials 24, no. 1 (2023): 305. 37131255 10.1186/s13063-023-07296-y PMC10155434

[42] C. McDermott , J. Vennik , C. Philpott , et al., “Maximising Recruitment to a Randomised Controlled Trial for Chronic Rhinosinusitis Using Qualitative Research Methods: The Macro Conversation Study,” Trials 22 (2021): 54. 33436031 10.1186/s13063-020-04993-w PMC7805190

[43] L. Morris , J. Dumville , S. Treweek , N. Miah , F. Curtis , and P. Bower , “Evaluating a Tool to Improve Engagement and Recruitment of Under‐Served Groups in Trials,” Trials 23, no. 1 (2022): 867. 36210444 10.1186/s13063-022-06747-2 PMC9549666

[44] NIHR “Improving Inclusion of Under‐Served Groups in Clinical Research: Guidance From the NIHR‐INCLUDE Project”. Vol. 2024. (NIHR, 2020), https://www.nihr.ac.uk/documents/improving-inclusion-of-under-served-groups-in-clinical-research-guidance-from-include-project/25435.

[45] “Census 2021,” Bristol City Council, accessed January 4, 2024, https://www.bristol.gov.uk/council-and-mayor/statistics-census-information/census-2021.

[46] “Payment Guidance for Researchers and Professionals,” National Institute for Health and Care Research, accessed September 2, 2023, https://www.nihr.ac.uk/documents/payment-guidance-for-researchers-and-professionals/27392.

[47] S. Staniszewska , J. Brett , I. Simera , et al., “GRIPP2 Reporting Checklists: Tools to Improve Reporting of Patient and Public Involvement in Research,” BMJ 358 (2017): j3453. 28768629 10.1136/bmj.j3453 PMC5539518

[48] D. Allen , L. Cree , P. Dawson , et al., “Exploring Patient and Public Involvement (PPI) and Co‐Production Approaches in Mental Health Research: Learning From the PARTNERS2 Research Programme,” Research Involvement and Engagement 6 (2020): 56. 32974052 10.1186/s40900-020-00224-3 PMC7507647

[49] D. H. Bodicoat , A. C. Routen , A. Willis , et al., “Promoting Inclusion in Clinical Trials—A Rapid Review of the Literature and Recommendations for Action,” Trials 22 (2021): 880. 34863265 10.1186/s13063-021-05849-7 PMC8643184

[50] J. Cunningham‐Erves , C. Barajas , T. L. Mayo‐Gamble , et al., “Formative Research to Design a Culturally‐Appropriate Cancer Clinical Trial Education Program to Increase Participation of African American and Latino Communities,” BMC Public Health 20 (2020): 840. 32493245 10.1186/s12889-020-08939-4 PMC7268329

[51] C. O. Ezeugwu , A. Laird , C. Daniel Mullins , D. S. Saluja , and R. A. Winston , “Lessons Learned From Community‐Based Minority Health Care Serving System Participation in an NIH Clinical Trial,” Journal of the National Medical Association 103, no. 9–10 (2011): 839–844. 22364051 10.1016/s0027-9684(15)30438-7

[52] M. E. Ford , L. A. Siminoff , E. Pickelsimer , et al., “Unequal Burden of Disease, Unequal Participation in Clinical Trials: Solutions From African American and Latino Community Members,” Health & Social Work 38, no. 1 (2013): 29–38. 23539894 10.1093/hsw/hlt001 PMC3943359

[53] C. Heller , J. E. Balls‐Berry , J. D. Nery , et al., “Strategies Addressing Barriers to Clinical Trial Enrollment of Underrepresented Populations: A Systematic Review,” Contemporary Clinical Trials 39, no. 2 (2014): 169–182. 25131812 10.1016/j.cct.2014.08.004 PMC6936726

[54] R. P. Amorrortu , M. Arevalo , S. W. Vernon , et al., “Recruitment of Racial and Ethnic Minorities to Clinical Trials Conducted Within Specialty Clinics: An Intervention Mapping Approach,” Trials 19 (2018): 115. 29454389 10.1186/s13063-018-2507-9 PMC5816509

[55] M. Coakley , E. O. Fadiran , L. J. Parrish , R. A. Griffith , E. Weiss , and C. Carter , “Dialogues on Diversifying Clinical Trials: Successful Strategies for Engaging Women and Minorities in Clinical Trials,” Journal of Women's Health 21, no. 7 (2012): 713–716. 10.1089/jwh.2012.3733 PMC3432572 22747427

[56] K. M. Sturgeon , R. Hackley , A. Fornash , et al., “Strategic Recruitment of an Ethnically Diverse Cohort of Overweight Survivors of Breast Cancer With Lymphedema,” Cancer 124, no. 1 (2018): 95–104. 28881471 10.1002/cncr.30935 PMC5743016

[57] C. Jameson , Z. Haq , S. Musse , Z. Kosar , G. Watson , and V. Wylde , “Inclusive Approaches to Involvement of Community Groups in Health Research: The Co‐Produced CHICO Guidance,” Research Involvement and Engagement 9, no. 1 (2023): 76. 37679854 10.1186/s40900-023-00492-9 PMC10486022

[58] C. McGrath , M. R. Kennedy , A. Gibson , S. Musse , Z. Kosar , and S. Dawson , “World Cafés s a Participatory Approach to Understanding Research Agendas in Primary Care With Underserved Communities: Reflections, Challenges and Lessons Learned,” Research Involvement and Engagement 9, no. 1 (2023): 101. 37898808 10.1186/s40900-023-00509-3 PMC10613381

[59] S. Dawson , K. Banister , K. Biggs , et al., “Trial Forge Guidance 3: Randomised Trials and How to Recruit and Retain Individuals From Ethnic Minority Groups—Practical Guidance to Support Better Practice,” Trials 23, no. 1 (2022): 672. 35978338 10.1186/s13063-022-06553-w PMC9383663

[60] A. Kurt , L. Semler , M. Meyers , B. G. Porter , J. L. Jacoby , and B. Stello , “Research Professionals' Perspectives, Barriers, and Recommendations Regarding Minority Participation in Clinical Trials,” Journal of Racial and Ethnic Health Disparities 4 (2017): 1166–1174. 28004355 10.1007/s40615-016-0322-0

