# Supplementary material for: Understanding Barriers and Facilitators for Ethnic Minority Groups to Audio Recording Recruitment Discussions in Clinical Trials: A Participatory Approach to Improving Informed Consent and Participation
Source: Health Expect. 2025 Mar 17;28(2):e70210. doi: 10.1111/hex.70210 (PMC11913728; doi:10.1111/hex.70210)
Supplement: Supplementary file 1 — Supporting information. [file HEX-28-e70210-s001.docx]

**Appendix I**

**GRIPP-2 Short Form**

| **Section and topic** | **Item** | **Reported on page no** |
| --- | --- | --- |
| 1: Aim    Report the aim of the study | Therefore, we aimed to understand the facilitators and barriers to consenting to audio-recording recruitment discussions within the clinical trial context, which in turn can influence consent to participation in trials. | 5 |
| 2: Methods    Provide a clear description of the methods used for patient and public involvement (PPI) in the study | To foster a co-creation process, this project included from its inception the active involvement of our PPIE collaborators and co-applicants for funding, 'Khaas'. Khaas is a community based third sector organisation that has worked across the Southwest and the Bristol area, delivering health, educational and social services to improve the lives of ethnic minorities and disabled children, their carers and families for over 35 years. They also helped us reach contributors from the South Asian community (mainly of Pakistani and Bangladeshi origin) and arrange workshops.  Our two PPIE contributors, Zahra Kosar and Samira Musse, helped us reach the Somali community via The Friday Coffee Morning, which is a weekly, community-run event at the Wellspring Settlement. The meeting gathers about 10-15 local women, predominantly of Somali background, to socialise. Similarly, the Chinese Community Wellbeing Society helped us reach people from the Chinese community. The description of workshops and contributors that attended the discussion can be seen in table 1.  [Insert Table 1 here]  Connecting with community leaders was an essential first step. The goal was to establish links and develop relationships to facilitate introductions and advocacy. Building rapport entailed direct in-person meetings, supplemented by phone calls and emails, to discuss preferred approaches to involvement activities. The discussion format and interactions were customised based on the needs and inclinations of each distinct community group, with open-ended, flexible conversations adapted to best suit different preferences. | 5-6 |
| 3: Results    Outcomes—Report the results of PPI in the study, including both positive and negative outcomes | The following six themes were identified in the discussions about consent to take part in a trial and audio-recording of recruitment discussion.  1. Building Trust in Research  The most common issue discussed during the workshops was how trust can be built with these communities during recruitment. Language emerged as a significant factor for trust building. Contributors believed that accessible information in their native language would be received well. They cautioned against relying solely on automated tools, so professional translators fluent in medical terminology were crucial to communicate concerns effectively.  ‘Translator is needed so I can explain my concern’ (Workshop 1, Somali men)  Using plain language and having community representatives were deemed essential for comprehension and confidence in recruitment discussions. Having people from the same cultural background on the trial team would also encourage participation.  ‘More people will join the trials once some people understand – more approachable if we already know people on the trial.’ (Workshop 4, South Asian men)    Moreover, Somali contributors were more hesitant when the trial involved children, especially as part of random allocation. This caused contributors to ask ‘why [am I] getting different treatment?’ which in their opinion increased their deep-rooted mistrust of research related to historical mistreatment. They referred to the Tuskegee experiment to explain their concern, even though they weren’t directly impacted by it, they remembered how their communities have been treated in the past.  Chinese community partners asked what insurance and liability are given if harm occurs, explaining insurance would increase their trust. They expected their recruiter to be honest with them about potential treatment risks, which would show their respect and care. For them, a convincing answer to ‘Why should I join?’ was grounded in trust, not monetary or any other personal gain. They mentioned that with care and understanding from researchers, sceptical questions can become an opportunity to build trust through dialogue.  2. Views on audio recording recruitment discussions  The discussion about audio recording of recruitment discussion varied among the three communities. The South-Asian women admitted to censoring or withholding their concerns and opinions when recorded (during mock discussion). Similarly, South-Asian men were not willing to openly discuss their personal health matters when recorded. They discussed their fear of misrepresentation in case someone hears part of the discussion without knowing the full context. Therefore, they mentioned that they would be very cautious in how they phrased their statements, which might interrupt a candid dialogue.  ‘My words may be misinterpreted – “only you know what you mean” i.e. it is possible to misspeak – if recorded you feel careful of what you say.’ (Workshop 4, South Asian men)  In contrast, Somali men were comfortable doing recorded discussions mainly in a one-to-one setting with doctors and nurses. Some community partners from Somali women mentioned: ‘Sound of my own voice is embarrassing.’ (Workshop 3). Therefore, they showed interest in receiving written notes or verbatim of the discussion for their record rather than the actual recording.  Unlike the other two community groups, most Chinese community members were quite open to audio recording, with most being comfortable if they were granted access to the recording afterwards.  ‘No major concerns about audio recording – though it is a good idea if patient is then given access to audio recording’ (Workshop 5, Chinese community member)  They reasoned that the provision of audio recording would demonstrate transparency and be suitable for record-keeping if they needed to recall what was discussed and agreed upon. Some mentioned that agreeing to audio recording is conditional to the value of the trial and the integrity of the trial team.  It was a common argument among all three groups that they were wary of how their recordings would be used and by whom beyond the recruiters. They needed clarity about handling of and access to recorded data.  3. Ethical safeguards  In the South Asian community discussion, it was highlighted that they feared the presence of an interpreter for translation as they might know them from the community and therefore there could be a breach of confidentiality of their recruitment discussion. Especially the older generation was sceptical about other people from the wider community knowing of their involvement in research. This was especially the case for health conditions which could be sensitive in nature. Both Somali and Chinese community members largely felt apprehensive about the selection method and worried they would be treated like ‘lab rats’ or ‘test subjects’. Therefore, understanding the trial focus, risks and benefits of treatment was highly important for them.  All community contributors stressed the importance of full transparency in trial procedures. They needed to know in advance what data would be collected, why and how their privacy would be safeguarded.  4. Missed opportunities  An interesting discussion emerged among the South Asian and Somali women. They discussed their frustration over the lack of outreach from the general practitioners (GPs) in encouraging or informing them about clinical trials. They argued that if GPs didn’t inquire about their interests in taking part in studies, this would limit their opportunity to get involved. Some defended the GPs by arguing that with increased demand on healthcare practitioners, there is limited time in the ’10 min slot’ to address ‘research’. They talked about their lack of awareness about trials relevant to them and their difficulty in finding information.  Some South-Asian women mentioned that they felt minimal effort has been made to include them in research, even when there is no language barrier, and they can communicate well in English.  ‘Language barrier for some people but we don’t get approached at all.’ (Workshop 2, South Asian women)  Overall, these women groups also highlighted some barriers to taking part in trials that were not mentioned in male groups, such as time constraints, the need to prioritise family matters, the voluntary nature of the study, childcare support, and transport.  5. Altruism as a motivating factor  Altruism was a common motivation found in all discussions. For the South Asian community, despite their religious concerns and uncertainties about experimental drug compositions, there was a commitment to support medical research for the greater good and the well-being of future generations. Similarly, Chinese and Somali communities also emphasised that they were interested in research that could help others and is meaningful. The common sentiment that echoed was:  ‘If it’s going to help someone then why not.’  6. Cultural Factors  Some unique cultural factors among each community highlighted their concerns in relation to participation in clinical trials and/or consenting to audio recording.  South Asian women voiced different concerns compared to men, which included a lack of independence in making decisions regarding their health and the significant influence of family members in decision-making. Some mentioned difficulties they would face in convincing men in their families if they wanted to participate, pointing out men's different priorities and mindsets compared to them. They were also concerned about certain medications and procedures which might not comply with their religious dietary restriction. Another added concern was the complexity of explaining traditional cultural practices to researchers.  Meanwhile, South Asian men highlighted issues related to gender. They acknowledged their discomfort in discussing topics of men’s health like prostate cancer screening with female physicians or researchers. We highlighted the issues raised by women in their discussion related to being dependant on families for their decisions. These men criticised their male peers and discouraged the influence of men on women's health decisions, stressing that women should have been independent in making their choice. Somali men and women advised aligning same-sex recruiter and translator when recruiting for sensitive subjects, whereas, for general health issues, the gender of the recruiter was not important. They accentuated that gender-based segregation aligns with their religious and cultural preferences.  ‘Generic topic who discusses does not matter. If it is a sensitive topic, match men with men and women with women. Same for translators as well.’ (Workshop 1, Somali men)  Due to stigma around certain issues like organ donation, they emphasised the need for guidance from their religious leaders to help them align their religious beliefs and values when participating in such trials. Candid discussion with the recruiters would also help in deciding how such trials fits with their religious and cultural beliefs.  In contrast, Chinese community partners discussed their culture rooted in Chinese family-based care, contrasting with the individual focus in the UK healthcare system. For them, discussing with their family was a priority before making any healthcare decision. Hence, involving family members in the recruitment discussion could be another facilitating approach.  ‘Family members communicate to direct care – it is difficult to get UK culture to accept family basis of care.’ (Workshop 5, Chinese community member)  Furthermore, the positive experiences of peers can highly influence decision-making. As in the South Asian community, in the Chinese community, reluctance from male family members could cause obstacles. However, they suggested that recruiters should recognise and accommodate Chinese communal decision-making.  7. Value of approach  From the discussions, it was evident that the contributors valued how they were approached, and this was related to a positive or negative response to trial recruitment.  For South Asian men, if the trial team approached them through community-trusted institutions or religious institutions (mainly mosques and Imams), the chances of their participation would increase. Because they were Muslim, South Asian contributors wanted reassurance about trial drug contents (such as non-Halal ingredients) and safety, which they thought the religious institutes could help them verify. They believed it would not matter whether the recruitment process was recorded if they were approached via a trusted organisation. For South Asian contributors, advertisements on Asian TV channels and explanatory videos could also boost understanding, as many find visuals easier than text, whereas, some preferred face-to-face interactions to build trust by assessing recruiters' sincerity.  For the Somali community, meeting people already participating in the trial would help in making decisions by having first-hand knowledge of good and bad experiences. They registered their resistance by saying, 'I am not a lab rat', and condemned being treated solely as a research subject. They emphasised being approached in a welcoming, respectful manner and establishing a sense of partnership. They wanted their recruiter or consent taker to know the subject matter so that they could get sufficient information and discuss the risks and benefits of the study.  For the Chinese community, information about the trial was most trusted if their GPs recommended it or if they were familiar with the trial team. The GP's assurance would ensure the trial's legitimacy and boost their confidence in participation. However, they disapproved of generic letters from the GPs, broad advertisements, and mass mailing.  ‘Usually I just throw away a letter from the GP for a trial.’ (Workshop 5, Chinese community member)  For them, personalised discussion was the preferred method of approach. They believed it would allow them to ask questions, understand the trial and build rapport with the team. | 8-13 |
| 4: Discussion    Outcomes—Comment on the extent to which PPI influenced the study overall. Describe positive and negative effects | For many of us authors, conducting PPIE was a new experience. However, with guidance from the experienced member, we ensured that trusting relationships were built with the PPIE partners, and a safe space was provided to the contributors for an open discussion.48  Across all these discussions, the major emerging theme was building trust via approaching community leaders or influencers. Lack of trust and limited access to trials among underserved groups in research were the barriers also identified in a rapid review by Bodicoat and colleagues.49 There have been several studies that have successfully used the strategy of engaging with communities to build trust by providing educational sessions for them.50-56 A PPIE study involving Bristol researchers and community organisations working with South Asian, African Caribbean, and Somali groups also emphasised the importance of building trust.57 Their co-produced CHecklist for Inclusive COmmunity involvement in health research (CHICO) highlights engaging community leaders and understanding their groups as essential first steps in PPIE.  Language and cultural sensitivity were other common emerging themes. People were keen to participate in clinical trials; however, they needed to be assured that their cultural preferences would be accommodated. Another PPIE study also emphasised the importance of cultural sensitivity, recommending that healthcare professionals and staff, receive cultural sensitivity training to account for cultural context in patient interactions.58 This key theme is also a recommendation in Trial Forge Guidance 3,59 which highlights importance of language, translation and cultural appropriateness when developing trial materials to promote inclusivity.  For those facing a language barrier, the presence of a professional interpreter during recruitment discussions was highly encouraged, especially when the discussions were to be audio recorded. Since audio recordings serve as evidence of the discussion, contributors wanted to be certain they completely understand every detail, without confusion due to language. A skilled interpreter could ensure comprehension of the given information. However, it should be noted that due to resource constraints, arranging interpreters is not always possible, and the language barrier continues to be the biggest barrier for many professional researchers in recruiting people from ethnic minorities.60  Furthermore, during our workshops, we needed to spend a substantial amount of time explaining a clinical trial to the contributors, as there were misconceptions and a lack of understanding in terms of what a trial involved. This task was crucial, as we needed it to be able to initiate the discussion. We had to clarify to the contributors what a clinical trial was and guide them through it. However, if we had a short video, presented in their language, which takes them through the process, it would have made the task much more straightforward. A preference for educational resources in the form of video was highlighted in the group discussions. Therefore, we created a video resource of approx. 5 min that can be used during other PPIE discussions or trial recruitment to answer basic questions related to clinical trials such as: what are clinical trials; why participate; diversity and inclusion; recruitment process; participants’ rights and IC; and reasons for audio recording recruitment discussion. It is free to use and available online at (https://t.co/9nGR5xd3Vr). The message in the video has been delivered by native speakers to resonate with the community.  These videos aim to serve as an educational tool to disseminate critical insights to a broader audience in future clinical trials, especially when working with diverse communities to facilitate research participation. When used in other PPIE events, this video can be cost-effective, as it has the potential to save time for individuals involved. Furthermore, it can help potential study participants make informed decisions when participating. These outputs can be shared via social media, the QuinteT website, TMRP groups, and future trial dissemination and PPI events with various community organisations. | 14-15 |
| 5: Reflections    Critical perspective—Comment critically on the study, reflecting on the things that went well and those that did not, so others can learn from this experience | There were some limitations of the project. It was difficult to recruit South Asian men. The reason could be working hours (as meetings were held during the day), lack of interest or weakness of our approach to reaching them. From South Asian community, all PPI male contributors were from British Pakistani ethnicity. Fewer Pakistani men attended because, while Khaas is predominant in the Pakistani community, it is run by women who, for cultural reasons, have less reach to men than women. For future engagement, it is important to consider approaching men through mosques or other cultural or religious places and hosting the event in the evening or on a non-working day to help in their recruitment . To better engage South Asian men in future PPIE work and facilitate wider research participation, it is crucial to educate existing male community leaders about PPIE, as current contributors are predominantly women and mosques only target men who attend them. We recommend that efforts be made to encourage inclusion of more male members of South Asian ethnicity in research discussions as their perception also influence the recruitment of women and kids in their family.  Other limitations include the inability to contact people who participated in the discussion again. As we wanted contributors to feel safe, no personal data were collected, which prevented us from reaching the same group for further discussion. Although there are eighteen minority groups in Bristol, due to the limited timeframe to complete the project, only the three largest groups were approached.  Our videos have been shared with the clinical trials community for project evaluation, and their feedback will be collected. Based on the feedback, we will focus on the impact and feasibility of the video in the recruitment process. | 15-16 |
